# Immune Checkpoint Molecules Expressed on CD4^+^ T Cell Subsets in Chronic Asymptomatic Hepatitis B Virus Carriers With Hepatitis B e Antigen-Negative

**DOI:** 10.3389/fmicb.2022.887408

**Published:** 2022-04-27

**Authors:** Dawei Cui, Daixi Jiang, Cuilin Yan, Xia Liu, Yan Lv, Jue Xie, Yu Chen

**Affiliations:** ^1^Department of Laboratory Medicine, The First Affiliated Hospital, Zhejiang University School of Medicine, Key Laboratory of Clinical In Vitro Diagnostic Techniques of Zhejiang Province, Institute of Laboratory Medicine, Zhejiang University, Hangzhou, China; ^2^Department of Blood Transfusion, The First Affiliated Hospital, Zhejiang University School of Medicine, Hangzhou, China; ^3^State Key Laboratory for Diagnosis and Treatment of Infectious Diseases, National Clinical Research Center for Infectious Diseases, Collaborative Innovation Center for Diagnosis and Treatment of Infectious Diseases, The First Affiliated Hospital, Zhejiang University School of Medicine, Hangzhou, China; ^4^ZJU-Hangzhou Global Scientific and Technological Innovation Center, Hangzhou, China

**Keywords:** hepatitis B virus, asymptomatic HBV carriers, CD4^+^ T cells, immune checkpoint molecules, chronic hepatitis B virus

## Abstract

**Background:**

Chronic hepatitis B virus (HBV) infection remains a major public health problem worldwide. Immune checkpoint molecules expressed on CD4^+^ T cells play critical roles in chronic HBV infection. However, their roles in chronic asymptomatic HBV carriers (ASCs) with hepatitis B e antigen (HBeAg)-negative remain unclear. In this study, we explored the role of immune checkpoint molecules expressed on CD4^+^ T cell subsets in chronic ASCs with HBeAg-negative.

**Methods:**

Human peripheral blood mononuclear cells (PBMCs) from the ASCs with HBeAg-negative and healthy controls (HC) were isolated, and immune checkpoint molecules expressed on CD4^+^ T cell subsets and serum cytokines were detected by flow cytometry. Moreover, the mRNA expressions of immune checkpoint molecules were analyzed by a real-time quantitative PCR assay.

**Results:**

In comparison with HC, CD4^+^ T cells highly expressed LAG-3, TIM-3, and PD-1 in PBMCs from chronic ASCs with HBeAg-negative. Interestingly, the expressions of TIM-3 and PD-1 on circulating follicular helper T (Tfh) cells in ASCs were significantly high. Moreover, high expressions of LAG-3, TIM-3, and PD-1 were different among Treg, Th1, Th2, and Th17 cells. In addition, the expressions of TIM-3 and CTLA-4 mRNA in PBMCs from ASCs were significantly elevated. However, the frequency of CTLA-4^+^CD4^+^ T cell subsets in PBMCs from ASCs was not different from HC. The levels of six cytokines in serum from ASCs were not clearly different from HC.

**Conclusion:**

Immune checkpoint molecules highly expressed on CD4^+^ T cell subsets indicated an important role in chronic ASCs with HBeAg-negative, which provided potential therapeutic targets in the pathogenesis of chronic HBV infection.

## Introduction

Hepatitis B virus (HBV) infection remains a major health problem in the world, which accounts for roughly one-third of the global population (Yuen et al., 2018; Revill et al., 2019; Iannacone and Guidotti, 2022). Although only fewer than 5% of adults with HBV infection can develop chronic hepatitis B (CHB), over 250 million individuals with about one million deaths annually are affected with persistent infection, liver cirrhosis, and hepatocellular carcinoma (HCC) worldwide (Trepo et al., 2014; Yuen et al., 2018; Revill et al., 2019). Moreover, the population with hepatitis B e antigen (HBeAg)-negative is dominant with over 85% in the hepatitis B surface antigen (HBsAg)-positive subjects, and more than 350 million are chronic HBV carriers. The chronic HBV carriers with HBsAg-positive and HBeAg-negative can also develop HCC and liver-associated mortality that are related to baseline age, sex, serum HBV-DNA copies, and alanine aminotransferase (ALT) level (Kumar et al., 2009; Trepo et al., 2014; Koffas et al., 2021). Chronic asymptomatic HBV carriers (ASCs) are major individuals (67–80%) among chronic HBV cases, who have normal levels of ALT and low HBV-DNA copies; although they are not treated commonly by drugs, a long-term follow-up for them is necessary (Kumar et al., 2009; Koffas et al., 2021). Although this “inactive” disease phase of the chronic ASCs with HBeAg-negative is closely associated with a favorable prognosis, it is not a truly “inactive carrier” with no obvious liver disease, and the reactivation and the potential risk of disease progression, including HCC and cirrhosis, are not negligible (Kumar et al., 2009; Puoti, 2013; Tang et al., 2018; Koffas et al., 2021). Therefore, these patients can still potentially develop liver disease for persistent HBV infection.

Chronic and persistent HBV infection is a critical causative agent for T-cell dysfunction, including CD4^+^ T and CD8^+^T cells that attenuate or lose the clearance of the virus. The dysfunction of T cells is closely associated with the upregulated immune checkpoint molecules, such as programmed cell death protein-1 (PD-1), lymphocyte activation gene-3 (LAG-3), T-cell immunoglobulin and mucin domain-3 (TIM-3), and cytotoxic T-lymphocyte antigen-4 (CTLA-4) (Bengsch et al., 2014; Park et al., 2016; Salimzadeh et al., 2018; Chen and Tian, 2019; Fisicaro et al., 2020; Ferrando-Martinez et al., 2021). Accumulating evidence indicates that the blockade of immune checkpoint molecules contributes to the restoration of dysfunctional T-cell-mediated adaptive immune responses, which partially promote the clearance of chronic viruses, including HBV, and the remission of the disease (Lang et al., 2019; Jin et al., 2021; Sears et al., 2021). The function of CD8^+^T cells can be impaired without the help of CD4^+^ T cells, and CD8^+^T cells contribute to controlling and clearing viruses (Zuniga et al., 2015; Saeidi et al., 2018; Sears et al., 2021). Unfortunately, the function of CD4^+^/CD8^+^T cells is dysregulated in CHB infection, which is closely correlated with high expressions of immune checkpoint molecules on them (Bertoletti and Ferrari, 2012; Dong et al., 2019; Buschow and Jansen, 2021). Therefore, the immune checkpoint molecules on specific CD4^+^ T cell subsets in chronic HBV infection should be further elucidated in the ASCs with HBeAg-negative, which will contribute to a better understanding of CD8^+^T cell dysfunction during CHB infection.

In this study, the immune checkpoint molecules (PD-1, TIM-3, LAG-3, and CTLA-4) expressed on specific CD4^+^ T cell subsets were investigated in peripheral blood mononuclear cells (PBMCs) from chronic ASCs with HBeAg-negative and normal ALT level, and the HBV-specific CD4^+^ T cell subset-associated cytokines were also detected, which will provide potential therapeutic targets for the development of chronic HBV infection.

## Materials and Methods

### Demographic Characteristics

A total of 22 chronic ASCs with HBeAg-negative and normal ALT levels were enrolled according to the Guideline of Prevention and Treatment for Chronic Hepatitis B (2019 version), and gender- and age-matched 22 healthy controls (HC) were recruited at the First Affiliated Hospital, Zhejiang University School of Medicine (Hangzhou, China) in this study. The individuals were excluded from the study if they have diabetes, autoimmune diseases, hematological system diseases, and other hepatotropic diseases, and the HC were also excluded if they have a vaccine history within 6 months. The ASCs with chronic HBeAg-negative and normal ALT levels who received HBV treatment within 6 months before blood sampling were also excluded. The clinical and laboratory characteristics of ASCs and HC are presented in Table 1. Written informed consent of all individuals was obtained according to the Declaration of Helsinki (1964) and was approved by the Medical Ethical Committee of the First Affiliated Hospital, Zhejiang University School of Medicine in this study (approval no. 2021-523).

**TABLE 1:** Demographic characteristics of chronic ASCs with HBeAg-negative and healthy controls.

Variables	ASCs (*n* = 22)	HC (*n* = 22)	Range of reference
Age (years)	47.64 ± 11.12	43.82 ± 7.24	
Gender (M/F)	14/8	14/8	
HBsAg (IU/mL)	1891.83 ± 430.27***	0.01 ± 0.01	0.00 - 0.05
HBeAg (S/CO)	0.41 ± 0.01	0.34 ± 0.01	≥1.00
Anti-HBc (S/CO)	7.89 ± 0.08***	2.11 ± 0.53	≥1.00
Anti-HBe (S/CO)	0.03 ± 0.01***	1.50 ± 0.11	<1.00
Anti-HBs (IU/L)	1.45 ± 0.73**	194.43 ± 61.57	≥10.00
ALT (U/L)	20.14 ± 1.82	21.36 ± 2.04	9 - 50
AST (U/L)	20.77 ± 1.36	18.23 ± 1.18	15 - 40
HBV-DNA (IU/mL)	<30	NA	LOD = 30

ASCs, symptomatic HBV carriers; HC, healthy controls; M/F, male/female; HBsAg, hepatitis B virus surface antigen; HBeAg, hepatitis B virus e antigen; ALT, alanine aminotransferase; AST, aspartate transferase; LOD, limitation of detection; NA, not applicable. ***P < 0.001.

### Serological Analysis of Hepatitis B Virus Markers and Liver Function

Serum ALT and AST levels were detected by an automated biochemical analyzer (Roche cobas p 671, Switzerland). The serum HBsAg, anti-HBs, HBeAg, anti-HBe, and anti-HBc levels were tested by a chemiluminescent microparticle immuno Assay (Abbott Alinity i, United States). The serum HBV-DNA titers were determined by an ABI 7500 fluorescent quantitative PCR system (Applied BioSystems, United States).

### Isolation of Human Peripheral Blood Mononuclear Cells

Human fresh PBMCs were isolated from fresh whole blood samples of the individuals by density gradient centrifugation using a Ficoll–Hypaque solution (CL5020, Netherlands) according to the manufacturer’s protocol. The isolated PBMCs were washed two times using sterile phosphate-buffered saline (PBS) and transferred into tubes for use in subsequent experiments.

### Flow Cytometry Analysis

Human PBMCs were resuspended with 200 μL PBS buffer and incubated with fluorochrome-conjugated antibodies, anti-human Krome Orange-CD4, Alexa Fluor 750-CD45RA, ECD-CD25 (Beckman Coulter Life Science, United States), Pacific blue™-CXCR5, PerCP/Cy5.5-PD-1, APC-TIM-3, Brilliant violet 650™-LAG-3, PE/Cy7-CTLA-4, FITC-CCR6, and PE-CXCR3 (BioLegend, United States), for 20 min at room temperature, and matched isotype controls were performed in this study according to the manufacturer’s instructions. The samples were detected by the CytoFLEX Flow Cytometer, and the data were analyzed by CyExpert software (Beckman Coulter, United States).

### Quantitative Real-Time Polymerase Chain Reaction

Total RNA was extracted from fresh PBMCs by the RNeasy Mini Kit (Qiagen, Germany), which was used to synthesize cDNA by a reverse transcription reagent kit (Takara, China). Real-time quantitative PCR was used to detect the expression levels of target genes in triplicate by Takara SYBR Supermix (Takara, China) by ABI QuantStudio 5 (Applied Biosystems, United States) as previously described (Shen et al., 2020). Glyceraldehyde 3-phosphate dehydrogenase (GAPDH) was used as an internal control in this study. The sequences of the specific primers are listed as follows: PD-1 forward 5′-AAGGCGCAGATCAAAGAGAGCC-3′ and reverse 5′-CAACCACCAGGGTTTGGAACTG-3′; TIM-3 forward 5′-GACTCTAGCAGACAGTGGGATC-3′ and reverse 5′-GGTGGTAAGCATCCTTGGAAAGG-3′; LAG-3 forward 5′-GCAGTGTACTTCACAGAGCTGTC-3′ and reverse 5′-AAGCCAAAGGCTCCAGTCACCA-3′; CTLA-4 forward 5′-ACGGGACTCTACATCTGCAAGG-3′ and reverse 5′-GGAGGAAGTCAGAATCTGGGCA-3′; and GAPDH forward 5′-GTCTCCTCTGACTTCAAC AGCG-3′ and reverse 5′-ACCACCCTGTTGCTGTAG CCAA-3′.

### Serological Detection of Cytokines

The serum levels of IFN-γ, IL-2, IL-4, IL-6, IL-10, and TNF-α were tested by the flow fluorescence immune microbeads assay (Celgene, China) according to the manufacturer’s protocol. In brief, 25 μL fluorescence detection reagent and 25 μL immune microbeads were added to 25 μL serum samples, and the mixed solutions were darkly incubated for 2.5 h at room temperature. Then, they were washed and resuspended with 100 μL PBS buffer, and cytokines were detected by BD FACSCanto II (BD, United States).

### Statistical Analysis

Statistical analysis of the experimental data was performed using the Mann–Whitney *U* test or a one-way ANOVA using GraphPad Prism 7 software (GraphPad Software, Inc., CA). The differences were determined between two unpaired groups by Student’s *t*-test, and significant differences were determined by paired *t*-tests between the two paired groups. Spearman’s correlation coefficients were used to analyze correlations of different variables between the two groups. All *P*-values < 0.05 were considered to be statistically different.

## Results

### Expanded Immune Checkpoint Molecules Expressed on CD4^+^ T Cells in Chronic Asymptomatic Hepatitis B Virus Carriers With Hepatitis B e Antigen-Negative

**FIGURE 1:**
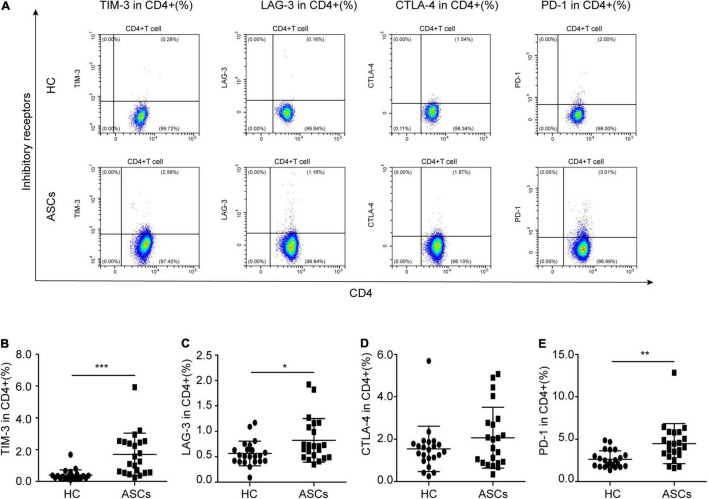
Immune checkpoint molecules expressed on CD4^+^ T cells in ASCs with HBeAg-negative. **(A)** Flow cytometry analysis of PD-1^+^, TIM-3^+^, LAG-3^+^, and CTLA-4^+^ on CD4^+^ T cells from the PBMCs of HC and ASCs. **(B)** The frequency of TIM-3^+^CD4^+^ T cells. **(C)** The frequency of LAG-3^+^CD4^+^ T cells. **(D)** The frequency of CTLA-4^+^CD4^+^ T cells. **(E)** The frequency of PD-1^+^CD4^+^ T cells. Data represent the mean± SD. *P< 0.05, **P< 0.01, and ***P< 0.001.

In this study, there was a significant difference in baseline characteristics, including HBsAg, anti-HBc, anti-HBe, and anti-HBs levels from the patients’ chronic ASCs with HBeAg-negative; however, no significant difference was found in ALT and AST levels between 22 chronic ASCs with HBeAg-negative and 22 healthy controls (HC) (Table 1). Previous reports indicated that circulating CD4^+^ T cells with immune checkpoint molecules played a critical role in chronic HBV infection, but the related studies about chronic ASCs were few (Ye et al., 2015; Dong et al., 2019). The frequencies of circulating CD4^+^ T cells with four immune checkpoint molecules, including TIM-3, LAG-3, CTLA-4, and PD-1, were detected between chronic ASCs with HBeAg-negative and HC by flow cytometry (Figure 1A). Compared with HC, the frequency of circulating TIM-3^+^CD4^+^ T cells, LAG-3^+^CD4^+^ T cells, and PD-1^+^CD4^+^ T cells was significantly elevated in ASCs (Figures 1B,C,E, respectively). However, the frequency of CTLA-4^+^CD4^+^ T cells was not significantly different between chronic ASCs with HBeAg-negative and HC (Figure 1D). In addition, there was no significant correlation between immune checkpoint molecules expressed on CD4^+^ T cells and serum HBsAg levels from chronic ASCs with HBeAg-negative (Table 2).

**TABLE 2:** The correlation of immune checkpoint molecules on CD4^+^ T cell subsets and serum HBsAg levels from 22 chronic ASCs with HBeAg-negative.

CD4^+^ T cell subsets	Spearman r	*P*-value	CD4^+^ T cell subsets	Spearman r	*P*-value
PD-1^+^CD4^+^ T	−0.08978	0.6911	PD-1^+^Th1	−0.3796	0.0815
LAG-3^+^CD4^+^ T	−0.1413	0.5306	LAG-3^+^Th1	−0.2243	0.3156
TIM-3^+^CD4^+^ T	−0.3134	0.1556	TIM-3^+^Th1	−0.2309	0.3011
CTLA-4^+^CD4^+^ T	−0.1293	0.5663	CTLA-4^+^Th1	−0.1870	0.4048
PD-1^+^Tfh	−0.1034	0.6471	PD-1^+^Th2	0.02654	0.9076
LAG-3^+^Tfh	−0.02598	0.9086	LAG-3^+^Th2	−0.1169	0.6042
TIM-3^+^Tfh	−0.2417	0.2784	TIM-3^+^Th2	−0.1982	0.3766
CTLA-4^+^Tfh	−0.1593	0.4789	CTLA-4^+^Th2	−0.07284	0.7473
PD-1^+^Treg	−0.2465	0.2687	PD-1^+^Th17	−0.1688	0.4526
LAG-3^+^Treg	−0.1914	0.3935	LAG-3^+^Th17	−0.07623	0.7360
TIM-3^+^Treg	−0.3800	0.0811	TIM-3^+^Th17	−0.2840	0.2002
CTLA-4^+^Treg	−0.09317	0.6801	CTLA-4^+^Th17	−0.07510	0.7398

### Immune Checkpoint Molecules Expressed on Tfh Cells in Chronic Asymptomatic Hepatitis B Virus Carriers With Hepatitis B e Antigen-Negative

**FIGURE 2:**
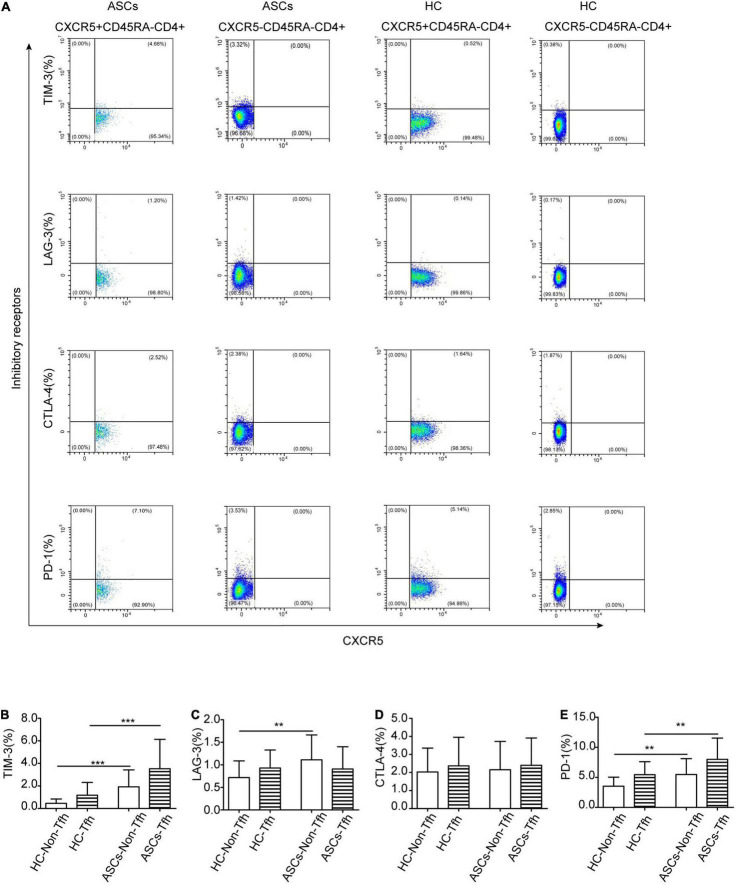
Immune checkpoint molecules expressed on CXCR5^+^CD45RA^–^CD4^+^ Tfh cells. **(A)** Flow cytometry detection of PD-1^+^, TIM-3^+^, LAG-3^+^, and CTLA-4^+^ on CXCR5^+^CD45RA^–^CD4^+^ Tfh cells and CXCR5^–^CD45RA^–^CD4^+^ Th cells in ASCs and HC. **(B–E)** Analysis of the expression of PD-1^+^, TIM-3^+^, LAG-3^+^, and CTLA-4^+^ on CXCR5^+^CD45RA^–^CD4^+^ Tfh cells and CXCR5^–^CD45RA^–^CD4^+^ Th cells in ASCs and HC. Data represent the mean± SD. **P< 0.01, ***P< 0.001.

Accumulated evidence showed the critical role of follicular helper T (Tfh) cells with high expressions of immune checkpoint molecules including TIM-3 and PD-1 in viral infection, including chronic HBV infection (Ye et al., 2015; Vella et al., 2017; Zhuo et al., 2017; Crotty, 2019; Yan et al., 2020; Cui et al., 2021; Liu et al., 2022). In this study, effector CD45RA^–^CXCR5^+^CD4^+^ Tfh cells with immune checkpoint molecules were detected by flow cytometry (Figure 2A). The frequency of circulating CD4^+^ T cells with immune checkpoint molecules was detected between chronic ASCs with HBeAg-negative and HC by flow cytometry (Figure 2A). Compared with HC, the frequency of circulating TIM-3^+^CD45RA^–^CXCR5^+^CD4^+^ Tfh cells and PD-1^+^CD45RA^–^CXCR5^+^CD4^+^ Tfh cells was notably increased, which also was higher than that of TIM-3^+^CD45RA^–^CXCR5^–^CD4^+^ T cells and PD-1^+^CD45RA^–^CXCR5^–^CD4^+^ T cells in ASCs (Figures 2B,E). However, there was no significant difference in the frequency of LAG-3^+^CD45RA^–^CXCR5^+^CD4^+^ Tfh cells CTLA-4^+^CD45RA^–^ CXCR5^+^CD4^+^ Tfh cells, TIM-3^+^CD45RA^–^CXCR5^–^CD4^+^ T cells, and PD-1^+^CD45RA^–^CXCR5^–^CD4^+^ T cells between chronic ASCs with HBeAg-negative and HC (Figures 2B–E). In addition, there was no significant correlation between immune checkpoint molecules expressed on Tfh cells and serum HBsAg levels from chronic ASCs with HBeAg-negative (Table 2).

### Immune Checkpoint Molecules Expressed on Treg Cells

**FIGURE 3:**
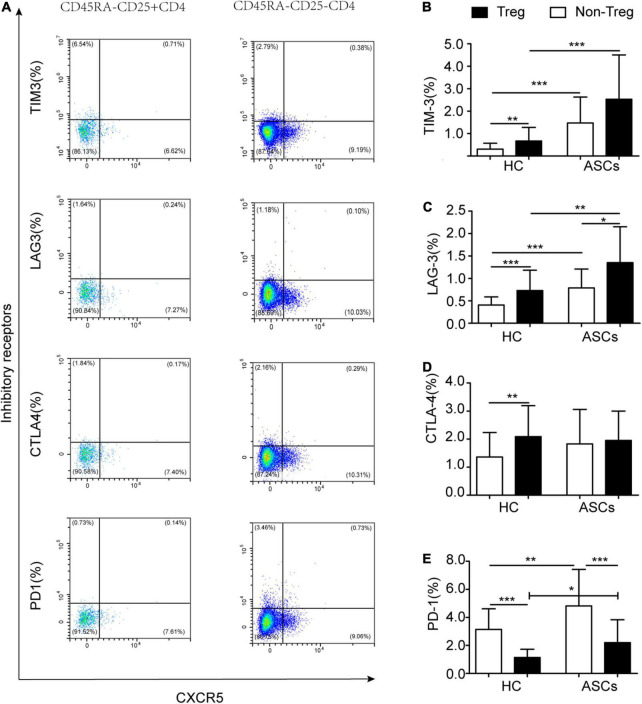
Immune checkpoint molecules expressed on CD45RA^–^CD25^+^CD4^+^ Treg cells in HBV carriers. **(A)** Flow cytometry detection of PD-1^+^, TIM-3^+^, LAG-3^+^, and CTLA-4^+^ on Treg cells. **(B–E)** Different expressions of PD-1^+^, TIM-3^+^, LAG-3^+^, and CTLA-4^+^ on Treg cells in ASCs and HC. Data represent the mean ± SD. *P< 0.05, **P< 0.01, and ***P< 0.001.

Previous reports indicated that Treg cells with high expressions of immune checkpoint molecules played an essential role in cancer types, autoimmune diseases, and viral infections (Andrews et al., 2019; Schnell et al., 2020; Granito et al., 2021). In this study, CD45RA^–^CD25^+^CXCR5^–^CD4^+^ Treg cells with immune checkpoint molecules were detected by flow cytometry (Figure 3A). In comparison with that of HC, the frequency of CD45RA^–^CD25^+^CXCR5^–^CD4^+^ Treg cells with TIM-3 or PD-1 and the frequency of LAG-3^+^CD45RA^–^CD25^+^CXCR5^–^CD4^+^ Treg cells in chronic ASCs with HBeAg-negative were significantly high (Figures 3B,C,E). Moreover, similar significant results were found for the frequency of CD45RA^–^CD25^–^CXCR5^–^CD4^+^ T cells with TIM-3 or PD-1 (Figures 3B,E). However, there was no significant difference in the frequency of CTLA-4^+^CD45RA^–^CD25^+^CXCR5^–^CD4^+^ Treg cells between chronic ASCs with HBeAg-negative and HC (Figure 3D). In addition, there was no significant correlation between immune checkpoint molecules expressed on Treg cells and serum HBsAg levels from chronic ASCs with HBeAg-negative (Table 2).

### Immune Checkpoint Molecules Expressed on Other CD4^+^ T Cell Subsets in Chronic Asymptomatic Hepatitis B Virus Carriers With Hepatitis B e Antigen-Negative

**FIGURE 4:**
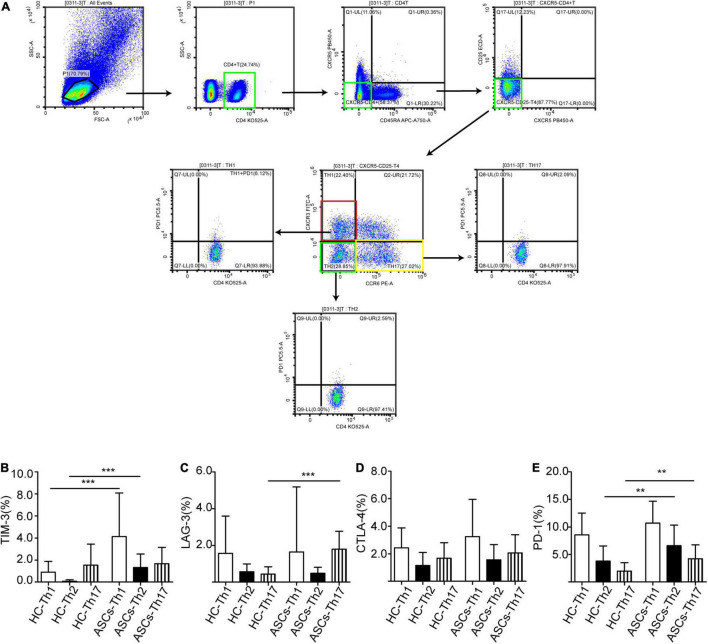
CD4^+^ T cell subsets showed different levels of immune checkpoint molecules. **(A)** Analysis of CD4^+^ T cell subsets. **(B)** TIM-3^+^ expression on Th1, Th2, and Th17 cells. **(C)** LAG-3^+^ expression on Th1, Th2, and Th17 cells. **(D)** CTLA-4^+^ cells expressed on Th1, Th2, and Th17 cells. **(E)** PD-1^+^ expression on Th1, Th2, and Th17 cells. Data represent the mean ± SD. **P< 0.01; ***P < 0.001.

Previous studies showed that human Th1 (CXCR3^+^CCR6^–^), Th2 (CXCR3^–^CCR6^–^), and Th17 (CXCR3^–^CCR6^+^) cells from PBMCs were distinguished based on the expressions of chemokine receptors CXCR3 and CCR6 on CD4^+^ T cells (Maecker et al., 2012; Kubo et al., 2017). In this study, to further investigate the frequency of immune checkpoint molecules expressed on other CD4^+^ T cell subsets in chronic ASCs with HBeAg-negative and HC, flow cytometry was used to detect these immune checkpoint molecules, and human Th1 (CXCR3^+^CCR6^–^), Th2 (CXCR3^–^CCR6^–^), and Th17 (CXCR3^–^CCR6^+^) cells were also defined by gating CD45RA^–^CXCR5^–^CD4^+^ T cells from PBMCs of ASCs and HC (Figure 4A). The results showed that the frequency of TIM-3^+^Th1 cells, TIM-3^+^Th2 cells, LAG-3^+^Th17 cells, PD-1^+^Th2 cells, and PD-1^+^Th17 cells was significantly increased; However, the frequency of TIM-3^+^Th17 cells, LAG-3^+^Th1 cells, LAG-3^+^Th2 cells, CTLA-4^+^Th1/2/17 cells, and PD-1^+^Th1 cells was not varied considerably in chronic ASCs with HBeAg-negative in comparison with HC (Figures 4B–E). Interestingly, Th1 cells highly expressed TIM-3, CTLA-4, and PD-1 compared with Th2 and Th17 cells (Figures 4B,D,E), Th1 and Th17 cells were highly expressed LAG-3 compared with Th2 cells in chronic ASCs with HBeAg-negative (Figures 4B,C). In addition, there was no significant correlation between immune checkpoint molecules expressed on Th1, Th2, and Th17 cells and serum HBsAg levels from chronic ASCs with HBeAg-negative (Table 2).

### Immune Checkpoint Molecules mRNA Expression in Chronic Asymptomatic Hepatitis B Virus Carriers With Hepatitis B e Antigen-Negative

**FIGURE 5:**
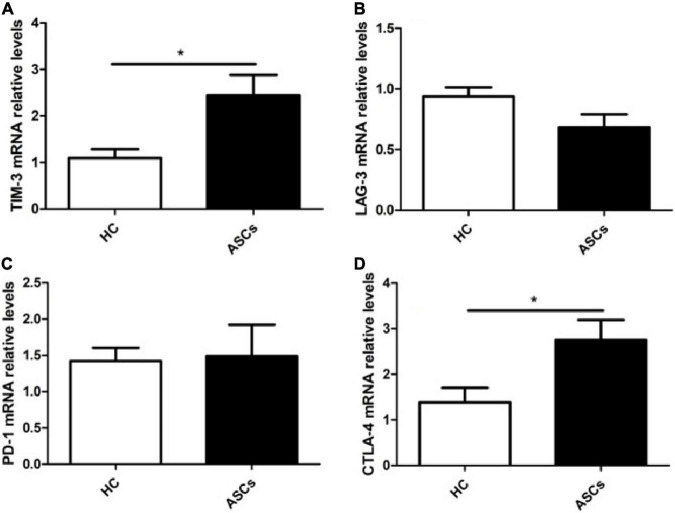
Expression levels of immune checkpoint molecules mRNA in PBMCs from chronic ASCs. **(A)** The relative levels of TIM-3 mRNA. **(B)** The relative levels of LAG-3 mRNA. **(C)** The relative levels of PD-1 mRNA. **(D)** The relative levels of CTLA-4. HC: *n* = 6, ASCs: *n* = 9. Data represent the mean ± SEM. *P< 0.05.

To further explore the mRNA levels of PD-1, TIM-3, LAG-3, and CTLA-4, the total RNA in PBMCs isolated from ASCs and HC was used to assess the expression levels of the four molecules by PCR method. The results displayed that the relative mRNA levels of TIM-3 and CTLA-4 genes were notably increased in the PBMCs from the ASCs in comparison with those from the HC (Figures 5A,D), but PD-1 and LAG-3 mRNA levels were not notably different between the ASCs and HC (Figures 5B,C).

### Serum Levels of Cytokines in Chronic Asymptomatic Hepatitis B Virus Carriers With Hepatitis B e Antigen-Negative

**FIGURE 6:**
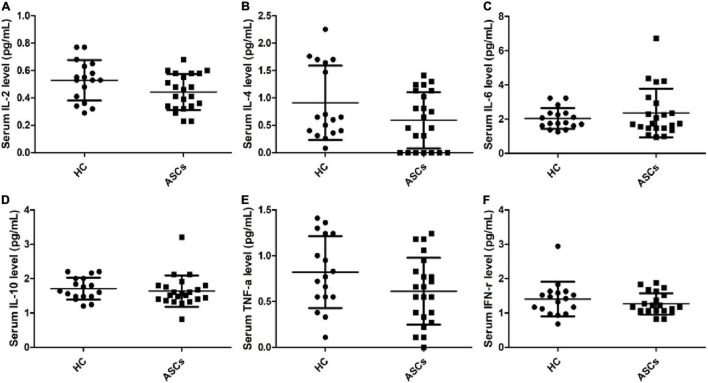
Expression levels of six cytokines in serum from chronic ASCs. **(A)** The relative levels of IL-2. **(B)** The levels of IL-4. **(C)** The levels of IL-6. **(D)** The relative levels of IL-10. **(E)** The relative levels of TNF-α. **(F)** The relative levels of IFN-γ. Data represent the mean ± SD.

To further investigate the immune status of ASCs with negative HBeAg, serum levels of some important cytokines were tested by flow cytometry. The results showed that decreased levels of serum IL-2, IL-4, and TNF-α were not notably different in chronic ASCs with negative HBeAg in comparison with HC (Figures 6A,B,E). Serum IL-6, IL-10, and IFN-γ levels were not significantly changed in chronic ASCs with HBeAg-negative in comparison with HC (Figures 6C,D,F). However, there was no correlation between these cytokine levels and HBsAg concentrations in chronic ASCs with HBeAg-negative (data not shown).

## Discussion

In this study, we provided an insight into the immune checkpoint molecules expressed on circulating human CD4^+^ T cell subsets from chronic ASCs with HBeAg-negative. The results showed that immune checkpoint molecules (TIM-3, LAG-3, and PD-1) expressed on CD4^+^ T cells were significantly upregulated in PBMCs from chronic ASCs with HBeAg-negative. Interestingly, a comprehensive analysis displayed a high frequency of circulating TIM-3^+^or PD-1^+^ Tfh cells in chronic ASCs with HBeAg-negative. Moreover, increased expressions of TIM-3, LAG-3, and PD-1 molecules on Th1, Th2, Th17, and Treg cells and TIM-3 and CTLA-4 mRNA levels in PBMCs from chronic ASCs with HBeAg-negative in comparison with HC were notably different. The chronic ASCs with HBeAg-negative can still develop into the stage of CHB with HBeAg-positive and end-stage liver disease including HCC and liver fibrosis (Kumar et al., 2009; Puoti, 2013; Tang et al., 2018; Koffas et al., 2021). Previous reports have shown that the co-inhibitory molecules on T cells contributed to the development of pathogenesis in patients with chronic HBV infection (Salimzadeh et al., 2018; Lang et al., 2019; Jin et al., 2021; Sears et al., 2021). Taken together, these findings indicated that elevated expressions of immune checkpoint molecules (TIM-3, LAG-3, and PD-1) on CD4^+^ T cell subsets might play a critical role in chronic ASCs with HBeAg-negative.

CD4^+^ T cell dysfunction in chronic HBV infection affected the function of other immune cells, including HBV-specific CD8^+^T cells and B cells, and resulted in disruption of the host’s immune responses and persistent HBV infection (Trepo et al., 2014; Yuen et al., 2018; Revill et al., 2019; Iannacone and Guidotti, 2022). Dysfunctional CD4^+^ T cells are closely correlated with the upregulation of immune co-inhibitory molecules expressed on CD4^+^ T cells that usually played an immunosuppressive function, such as TIM-3, PD-1, LAG-3, and CTLA-4 (Park et al., 2016; Salimzadeh et al., 2018; Dong et al., 2019; Fisicaro et al., 2020; Ferrando-Martinez et al., 2021). Previous reports showed an increased frequency of PD-1^+^CD4^+^ T cells, TIM-3^+^CD4^+^ T cells, LAG-3^+^CD4^+^ T cells, and CTLA-4^+^CD4^+^ T cells in chronic HBV infection, and the blockade of the co-inhibitory receptors contributed to the restoration of CD4^+^ T cell proliferation and function and promoted cytotoxic effector of CD8^+^T cells that played critical roles in viral clearance and disease pathogenesis of sustained HBV infection (Peng et al., 2008; Dong et al., 2017, 2019; Buschow and Jansen, 2021; Xiong et al., 2021). However, these studies did not display detailed data on ASCs of patients with chronic HBV. Our results showed an expanded frequency of PD-1^+^CD4^+^ T cells, TIM-3^+^CD4^+^ T cells, and LAG-3^+^CD4^+^ T cells, but not CTLA-4^+^CD4^+^ T cells in chronic ASCs with HBeAg-negative in comparison with HC. The number and status of the patients possibly affected the statistical analysis of CTLA-4^+^CD4^+^ T cell frequencies in this study. In addition, the mRNA levels of TIM-3 and CTLA-4 expressions, but not PD-1 and LAG-3 mRNA, were notably increased in chronic ASCs with HBeAg-negative in comparison with HC, which were not completely consistent with previous reports (Yu et al., 2009; Zhang et al., 2014; Cheng et al., 2021; Liu et al., 2021). The discrepancy may be associated with the number and the status of the special population who are the “inactive carrier” phase of chronic HBV infection, and these co-inhibitory receptors are also expressed in other immune cells, including CD8^+^T cells, innate lymphoid cells (ILCs), and NK cells (Kumar et al., 2009; Puoti, 2013; Riva and Chokshi, 2018; Tang et al., 2018; Mariotti et al., 2019; Fisicaro et al., 2020; Koffas et al., 2021; Mak et al., 2021).

Accumulating evidence indicates that high expressions of immune checkpoint molecules (TIM-3, PD-1, CTLA-4, and LAG-3) on CD4^+^ T cells are related to decreased secretion of cytokines and proliferation capacity of T cells, such as IFN-γ^+^Th1, IL-4^+^Th2, IL-17A^+^Th17, and IL-21^+^Tfh cells, except for PD-1*^high^* Tfh cells that have the capacity of inducing B-cell response and the generation of specific antibodies against antigens or viruses (Gibney et al., 2016; Park et al., 2016; Saeidi et al., 2018; Buschow and Jansen, 2021). However, Treg cells with increased expressions of immune checkpoint molecules displayed a high immunosuppressive function on HBV-specific Th1 and Tfh responses and promoted IL-10 and TGF-ß1 secretion (Stoop et al., 2005; Buschow and Jansen, 2021). This study showed that the frequencies of circulating TIM-3^+^or PD-1^+^ Tfh cells and TIM-3, LAG-3, and PD-1 molecules on Th1, Th2, Th17, and Treg cells were significantly expanded in chronic ASCs with HBeAg-negative in comparison with HC, which were partially accordant with previous reports (Stoop et al., 2005; Wang R. et al., 2018; Wang X. et al., 2018). These findings implied that chronic HBV infection resulted in the host’s immune disorder and dysregulation by upregulating immune checkpoint molecules, including TIM-3, PD-1, CTLA-4, and LAG-3 on T cells, that induced T-cell dysfunction in chronic HBV infection. In addition, our results showed that serum cytokines (IL-2, IL-4, and TNF-a) had a low trend in chronic ASCs with HBeAg-negative in comparison with HC, which were partially consistent with previous reports (Ayada et al., 2006; Wang X. et al., 2018; Zhong et al., 2021). These cytokines play a critical role in regulating CD4/CD8^+^T cell proliferation and differentiation and the production of various specific antibodies to control persistent HBV infection (Buschow and Jansen, 2021; Mak et al., 2021; Zhong et al., 2021).

Our study showed some interesting results, including four important immune checkpoint molecules expressed on CD4^+^ T cells and their subsets in chronic ASCs with HBeAg-negative. However, several limitations are listed in the following. First, the sample size was relatively small. Second, the function of these CD4^+^ T cells and their subsets with high expressions of the immune checkpoint molecules should be explored by a large number of samples in further study. The function of the co-inhibitory molecules on CD4^+^ T cells includes the cellular metabolism, cytokines secretion, differentiation, and helper capacity for CD8^+^T cells and B cells, which will be precisely explored by multi-omics methods.

## Conclusion

In summary, during chronic ASCs with HBeAg-negative infection, CD4^+^ T cells and their subsets, including Th1, Th2, Th17, Tfh, and Treg cells, showed high expressions of immune checkpoint molecules (TIM-3, PD-1, CTLA-4, and LAG-3) and decreased the secretion of cytokines. Currently, immune checkpoint-based therapies were used in many diseases, including cancer types, autoimmune diseases, and viral infections, which showed satisfactory curative effects for patients and animals. Taken together, immune checkpoint-based therapies have potential values for chronic ASCs with HBeAg-negative. These findings could provide potential therapeutic implications for chronic HBV infection by regulating CD4^+^ T cells’ functional therapy.

## Data Availability Statement

The original contributions presented in the study are included in the article, further inquiries can be directed to the corresponding author/s.

## Ethics Statement

The studies involving human participants were reviewed and approved by the Medical Ethical Committee of the First Affiliated Hospital, Zhejiang University School of Medicine (Approval No. 2021-523). The patients/participants provided their written informed consent to participate in this study. Written informed consent was obtained from the individual(s) for the publication of any potentially identifiable images or data included in this article.

## Author Contributions

DC wrote the manuscript. DC and DJ performed the experiments and analyzed the data. DC, DJ, CY, and YL collected the clinical data and samples. DC, DJ, and XL revised the manuscript. DC, YC, and JX conceived the topic and revised the manuscript. All authors approved the submitted version.

## Conflict of Interest

The authors declare that the research was conducted in the absence of any commercial or financial relationships that could be construed as a potential conflict of interest.

## Publisher’s Note

All claims expressed in this article are solely those of the authors and do not necessarily represent those of their affiliated organizations, or those of the publisher, the editors and the reviewers. Any product that may be evaluated in this article, or claim that may be made by its manufacturer, is not guaranteed or endorsed by the publisher.

## Abbreviations

HBV: hepatitis B virus
ASC: asymptomatic hepatitis B virus carriers
HC: healthy controls
PBMCs: peripheral blood mononuclear cells
Tfh: follicular helper T cells
Treg: regulatory T cells
PD-1: programmed cell death protein-1
TIM-3: T-cell immunoglobulin and mucin domain-3
CTLA-4: cytotoxic T-lymphocyte antigen-4
LAG-3: lymphocyte activation gene-3
IFN- γ: interferon-gamma
TNF- α: tumor necrosis factor- α
CXCR5: C-X -C motif chemokine receptor type 5
Th1: T helper cell type 1
Th2: T helper cell type 2
Th17: T helper cell type 17
HBeAg: hepatitis B e antigen
HCC: hepatocellular carcinoma
ALT: alanine aminotransferase
AST: aspartate aminotransferase
HBsAg: hepatitis B surface antigen
CCR6: C–C motif chemokine receptor 6
CXCR3: C–X–C motif chemokine receptor 3.
